# Pulmonary Actinomycosis Causing an Unusual Presentation in a Patient with COPD: A Case Report

**DOI:** 10.4314/ejhs.v35i3.10

**Published:** 2025-05

**Authors:** Ibrahim Nagmeldin Hassan, Muhsin Nagmeldin Hassan Ibrahim

**Affiliations:** 1 University of Khartoum, Faculty of Medicine, Khartoum, Sudan; 2 Sudan University of Science and Technology, Faculty of medicine Khartoum, Khartoum, SD

**Keywords:** Pulmonary actinomycosis, Thoracic actinomycosis, COPD, Pleural effusion, Sulfur granule

## Abstract

We present the case of a 63-year-old male patient with a history of chronic obstructive pulmonary disease (COPD), heavy smoking, and poor dental hygiene, who presented with progressive dyspnea, fever, and a productive cough. The patient was initially evaluated for pneumonia, but a chest radiograph revealed a right-sided pleural effusion. Further analysis of the pleural fluid showed an exudative effusion. Histopathological examination of a pleural biopsy sample identified gram-positive branching filamentous rods with yellow sulfur granules, consistent with a diagnosis of pulmonary actinomycosis. The patient was initially treated with intravenous amoxicillin/sulbactam, later switched to oral amoxicillin. This case highlights a rare clinical presentation of pleural effusion in a patient with pulmonary actinomycosis.

## Introduction

Actinomycosis is a chronic granulomatous infection caused by the gram-positive anaerobic bacterium Actinomyces, which forms long, branching filaments resembling fungal hyphae [1,2]. Actinomyces is part of the normal flora of the human mouth [1,2]. Actinomycosis can be classified into different clinical forms based on the site of infection: cervicofacial (the most common), abdominopelvic, and pulmonary. Pulmonary actinomycosis accounts for only about 15% of all cases [2]. It is more common in men in their fourth and fifth decades of life (with a male-to-female ratio of ≥3:1), and smokers with poor dental hygiene are at an increased risk [3]. The clinical and radiological features of pulmonary actinomycosis can resemble those of pneumonia, tuberculosis, and malignancy, often leading to misdiagnosis and delays in treatment [1,2]. Pulmonary actinomycosis can present with pleural effusion, which is a rare but notable manifestation in patients with COPD, as demonstrated in this case. This article reports a rare case of pulmonary actinomycosis in a COPD patient, presenting with an unusual clinical manifestation of pleural effusion.

## Case Presentation

A 63-year-old male presented with a two-week history of right-sided pleuritic chest pain, shortness of breath, high-grade fever, and a productive cough. He denied hemoptysis, night sweats, or weight loss. His medical history was significant for COPD, and he had been a heavy smoker (3–4 packs per day for at least 27 years) but had recently quit. He also reported being a heavy alcohol drinker but denied any drug use. He had no history of trauma, surgery, or recent travel.

Upon examination, his vital signs were as follows: a body temperature of 38.6°C, a heart rate of 100 beats per minute, a respiratory rate of 24 breaths per minute, and oxygen saturation of 90% on room air. He appeared thin and wasted but was not jaundiced or cyanotic. Chest examination revealed reduced chest expansion on the right side, a stony dull percussion note at the right base, and diminished breath sounds with a few crackles and wheezes. Oral examination revealed poor dentition. Cardiac and abdominal examinations were unremarkable, and no lymphadenopathy was noted.

**Figure 1:**
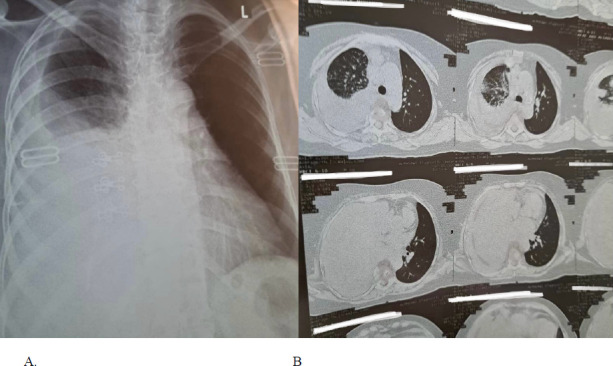
(A) Chest radiograph revealed a right-sided pleural effusion; (B) chest computed tomography was done to rule out any masses

Laboratory results showed a white blood cell count of 17,400/mm^3^ with neutrophilia (90%), hemoglobin of 11.5 g/dL, and an elevated C-reactive protein (CRP) level of 30.7 mg/dL. Renal function tests and electrolytes were normal, as were liver function tests. Chest X-ray revealed a right-sided pleural effusion, and chest computed tomography was performed to rule out masses (Figure 1). Pleural fluid was aspirated and analyzed, revealing a straw-colored exudative effusion with elevated protein and lactate dehydrogenase (LDH) levels. Pleural fluid analysis also showed a white blood cell count of 2930/µL, with 61.5% neutrophils and 37% lymphocytes. The pleural fluid adenosine deaminase (ADA) level was 36.7 IU/L. Cytological examination revealed inflammatory cells, but no microorganisms or malignant cells were identified. Ultrasound-guided percutaneous pleural biopsy was performed, and histological examination of the biopsy, along with microbiological culture, revealed inflammatory cell infiltrates with yellow sulfur granules and gram-positive branching filamentous rods, consistent with pulmonary actinomycosis. HIV testing was negative.

The patient was initially treated with intravenous amoxicillin/sulbactam (3 g every 8 hours) for two weeks, followed by oral amoxicillin for six months. A drainage chest tube was inserted to remove all pleural fluid until dryness. A dental consultation was arranged to address the patient's oral hygiene. Follow-up at 8 months post-discharge showed significant improvement in symptoms and resolution of radiological findings.

## Discussion

This case describes pulmonary actinomycosis in a patient with COPD, heavy smoking, and poor dental hygiene. Actinomycosis is a rare but important differential diagnosis in patients presenting with symptoms similar to more common respiratory conditions, such as pneumonia or tuberculosis. The patient's presentation with pleuritic chest pain, fever, productive cough, and dyspnea initially suggested pneumonia, making pulmonary actinomycosis a challenging diagnosis. Physicians must maintain a high index of suspicion, particularly in patients with risk factors such as smoking and poor oral hygiene. Early diagnosis can significantly improve patient outcomes, as delayed treatment can lead to complications like pleural effusion or empyema [5,6].

Actinomyces is a commensal organism that colonizes the human oral cavity. Pulmonary actinomycosis typically results from aspiration of oropharyngeal secretions, leading to direct invasion of the bronchopulmonary tree, often affecting the lower segments of the right lung, especially in individuals with poor dental hygiene or alcoholism [4]. Other predisposing factors include chronic lung conditions, such as chronic bronchitis, emphysema, and bronchiectasis [5,9].

Radiological features of pulmonary actinomycosis are nonspecific and may include consolidation, cavitation, abscess formation, draining sinuses, masses, and lymph node enlargement. Pleural involvement may result in pleural effusion, thickening, or empyema in approximately 15%–50% of cases [6]. These varied findings often lead to misdiagnosis as lung malignancy or tuberculosis. For instance, Song et al. [9] found that pulmonary actinomycosis frequently mimics these conditions, complicating diagnosis.

The diagnosis of pulmonary actinomycosis is challenging due to the difficulty of isolating the organism. Sputum culture or bronchoalveolar lavage (BAL) are usually inadequate unless cavitation is present. The gold standard is histopathological examination and anaerobic bacterial culture from pleural biopsy, looking for gram-positive branching filamentous rods with yellow sulfur granules [1-3]. Although sulfur granules are pathognomonic for actinomycosis, they can also be seen in nocardiosis, coccidioidomycosis, and aspergillosis, further complicating diagnosis [8]. Cultures from pleural effusion are often negative, making histological examination essential [4].

Treatment for pulmonary actinomycosis requires a prolonged course of high-dose beta-lactam antibiotics, such as penicillin G, amoxicillin, or cephalosporins, for 6–12 months.

The initial phase involves intravenous antibiotics for 2–6 weeks, followed by oral antibiotics [3-5]. Alternative options for penicillin-allergic patients include clindamycin, doxycycline, and erythromycin, with erythromycin being safe for pregnant women [1-3]. Surgical intervention may be necessary for patients with complications such as hemoptysis, empyema, abscesses, or sinus tracts unresponsive to antibiotics [9].

In conclusion, pulmonary actinomycosis should be considered in patients with COPD, particularly those with poor dental hygiene and a history of smoking, who present with nonspecific symptoms like fever, cough, and pleuritic chest pain. The diagnosis is challenging and requires a high index of suspicion. Early recognition, appropriate antibiotic treatment, and long-term follow-up are essential for favorable outcomes in these patients.
